# Neuronal Stress and Injury Caused by HIV-1, cART and Drug Abuse: Converging Contributions to HAND

**DOI:** 10.3390/brainsci7030025

**Published:** 2017-02-23

**Authors:** Ana B. Sanchez, Marcus Kaul

**Affiliations:** 1 Immunity and Pathogenesis Program, Infectious and Inflammatory Disease Center, Sanford Burnham Prebys Medical Discovery Institute, La Jolla, CA 92037, USA; asanchez@SBPdiscovery.org; 2 Department of Psychiatry, University of California San Diego, San Diego, CA 92093, USA

**Keywords:** HIV-1, HAND, anti-retroviral, methamphetamine, neurotoxicity

## Abstract

Multiple mechanisms appear to contribute to neuronal stress and injury underlying HIV-associated neurocognitive disorders (HAND), which occur despite the successful introduction of combination antiretroviral therapy (cART). Evidence is accumulating that components of cART can itself be neurotoxic upon long-term exposure. In addition, abuse of psychostimulants, such as methamphetamine (METH), seems to compromise antiretroviral therapy and aggravate HAND. However, the combined effect of virus and recreational and therapeutic drugs on the brain is still incompletely understood. However, several lines of evidence suggest a shared critical role of oxidative stress, compromised neuronal energy homeostasis and autophagy in promotion and prevention of neuronal dysfunction associated with HIV-1 infection, cART and psychostimulant use. In this review, we present a synopsis of recent work related to neuronal stress and injury induced by HIV infection, antiretrovirals (ARVs) and the highly addictive psychostimulant METH.

## 1. Introduction

Thirty-five years ago infection with human immunodeficiency virus (HIV-1) and acquired immunodeficiency syndrome (AIDS) evolved into an acute epidemic [1,2]. However, the development and use of combination antiretroviral therapy (cART) since the mid-1990s has changed the course of HIV infection/AIDS, which is now considered a chronic and treatable disease, if therapy is accessible [3]. Still, each year, more than 1 million people die from AIDS-related causes and 2.1 million people become newly infected by HIV-1. Globally, an estimated 36.7 million persons were living with HIV/AIDS in 2015, including more than 1.2 million people in the USA alone [4,5,6]. HIV-infection of the central nervous system (CNS) often leads to neurological problems and HIV-associated neurocognitive disorders (HAND) [7]. The underlying neuropathological mechanisms in humans are not completely understood and persist despite the use of antiretrovirals (ARVs) and effective virological control [7,8,9,10,11]. A major comorbidity of HIV infection is the abuse of drugs, such as heroin, cocaine and methamphetamine (METH), which is a public health problem in its own right [12]. Therefore, opiates, cocaine and METH are all being intensely studied in order to better understand their effects on HIV infection and HAND [13,14,15,16,17,18,19], but for the purpose of this review we will focus on the role of METH as one example of a widely abused drug. As such, HIV infection is frequently linked with the recreational use of the psychostimulant METH [20,21,22] and diminished adherence to cART regimens [23]. Additionally, increased viral loads have been linked to METH use in ART–receiving HIV positive individuals. [23,24]. The combination of METH and HIV-1 appears to trigger more neurocognitive impairment and neuropathology than either agent alone but the presumed mechanistic interaction of virus, anti-viral treatment and the psychostimulant drug is incompletely understood [25,26,27,28].

Clearly, the incidence of HIV-associated dementia (HAD), the most severe form of HAND, has declined with the implementation of cART [29,30], but the prevalence of cognitive impairment milder than dementia remains high in HIV patients on cART [9,11,31]. In addition, recent clinical studies observed that discontinuation of cART in virologically controlled HIV patients unexpectedly resulted in significant improvement of neurocognitive function [32,33,34,35]. Furthermore, experimental evidence is accumulating that at least some ARV compounds may themselves have neurotoxic effects [36,37,38,39,40]. These observations raised the possibility that certain ARVs may have neurotoxic effects that could contribute to the development of HAND.

Therefore, METH-using HIV patients are at risk of exposure to a combination of potential contributors to neurotoxicity: HIV and its components, a psychostimulant drug and ARVs. Here we will discuss current information about those contributing factors, the combination of which is being encountered in the clinical setting while an understanding of the mechanisms at the cellular level is only beginning to emerge.

## 2. HIV Infection, Neurotoxicity and HAND

Humans of all ages infected with HIV-1 develop neurological symptoms that include motor and cognitive dysfunction which are now termed HIV-associated neurocognitive disorders (HAND) [7,41,42,43]. HAND defines three categories of disorders with increasing severity according to standardized measures of dysfunction: (i) asymptomatic neurocognitive impairment (ANI); (ii) mild neurocognitive disorder (MND) and (iii) HAD. With the advent of cART, the incidence of HAND’s most severe form, dementia, decreased, suggesting a beneficial effect on cognitive function [29,30,44]. However, the prevalence of milder cognitive impairment (ANI, MND) remains high in HIV patients on cART, and HAND/HAD continues to be a significant independent risk factor for death due to AIDS [9,11,31,45]. Although improved control of peripheral viral replication and the treatment of opportunistic infections continue to extend survival times, cART fails to provide protection from HAND, or to reverse the disease in most cases [46,47,48,49,50,51]. One study found that in a group of 669 HIV patients who passed away between 1996 and 2001 more than 90% had developed HAD as an AIDS-defining condition during the last 12 months of life [52]. Moreover, the proportion of new cases of HAND/HAD displaying a cluster of differentiation 4^+^ (CD4^+^) T cell count greater than 200 μL^−1^ is growing [50]. This situation and distinct patterns of viral drug resistance in plasma and cerebrospinal fluid (CSF) compartments might at least in part be explained by limited penetration into the CNS of HIV protease inhibitors and several of the nucleoside analogues [46,53,54]. Therefore, it is possible that as people live longer with HIV-1 infection the prevalence of dementia may continue to rise [43,46,50,53,54,55,56].

Pathological features of HIV-1 infection in the brain are often referred to as HIV encephalitis (HIVE) and include activated resident microglia, microglial nodules, multinucleated giant cells, infiltration predominantly by monocytoid cells, including blood-derived macrophages, and decreased synaptic and dendritic density, combined with selective neuronal loss, widespread reactive astrocytosis, and myelin pallor [57,58]. However, the pathologic features best correlating with *ante mortem* measures of cognitive dysfunction include increased numbers of microglia [59], decreased synaptic and dendritic density, selective neuronal loss [58,60,61], elevated tumor necrosis factor (TNF)-α mRNA in microglia and astrocytes [62], and evidence of excitatory neurotoxins in CSF and serum [63]. Furthermore, two reports provide evidence that the amount of proviral HIV DNA in circulating monocytes and macrophages correlates better than viral load with the risk of developing HAD [64,65].

HIV infection can be associated with neuronal damage and loss in distinct brain regions, including frontal cortex [66,67], substantia nigra [68], cerebellum [69], and putamen [70] and features of neuronal apoptosis have been found in brains of HAD patients [71,72,73]. Moreover, the localization of apoptotic neurons was correlated with signs of structural damage and closely associated with evidence of microglial activation, especially within subcortical deep gray structures [71].

With the introduction of cART, HIV neuropathology began to shift. Although the incidence of opportunistic infections seemed to decline, two *post mortem* studies observed increased macrophage/microglia infiltration and activation in hippocampus and basal ganglia of cART-treated HIV patients as compared to samples from the pre-cART era as well as a higher prevalence of HIVE at the time of autopsy [25,74]. Specimens from HIV patients who had failed cART displayed even more encephalitis and severe leukoencephalopathy [74]. In line with these reports are more recent neuropathological descriptions of various forms with severe HIVE and white matter injury, extensive perivascular lymphocytic infiltration, “burnt-out” forms of HIVE and seemingly aging-related beta-amyloid accumulation implying an Alzheimer’s-like neuropathology [75,76].

HIV-1 appears to reach the brain soon after infection in the periphery, and then localizes primarily to perivascular macrophages and microglia [77,78,79,80]. Infection by HIV-1 of macrophages and lymphocytes in the periphery and microglia in the brain occurrs after the viral envelope protein gp120 binds to CD4 in conjunction with at least one of several possible chemokine receptors. Depending on the viral strain, different HIV-1 variants use CC chemokine receptor 5 (CCR5, CD195) and CCR3, or CXC chemokine receptor 4 (CXCR4, CD184), or a combination of these chemokine receptors to enter target cells [81,82,83]. Neurons and astrocytes in the brain also express chemokine receptors, including CCR5 and CXCR4 [84,85]. However these cells, in contrast to microglia, appear to be largely refractory to productive HIV-1 infection under in vivo conditions. However, several in vitro studies strongly suggest that CXCR4 is prominently involved in HIV-associated neuronal damage whereas CCR5 may play a dual role by being able to either serve a toxic or protective function [86,87,88,89,90,91,92]. Intact HIV-1, as well as picomolar concentrations of isolated viral envelope gp120, can induce neuronal death via CXCR4 and CCR5 receptors in neurons from humans and rodents [86,87,89,90,93,94,95,96,97,98].

While progress is being made in characterizing the neuropathological processes, how exactly HIV-1 infection provokes neuronal injury and death as well as neurocognitive and motor deficits remains controversial [43,53,54,79,99]. While it is generally agreed upon that HIV-1 does not infect post-mitotic, mature neurons, the mechanism of neuronal damage is a matter of debate and continuing investigation. Ample evidence exists that various viral proteins; including Tat, Nef, Vpr and the Env proteins gp120 and gp41, can initiate neuronal injury and death [43,53,93,99,100,101,102,103,104]. Moreover, we and others found more recently that HIV-1 and at least its gp120 can also compromise neurogenesis [8,105,106]. All these observations, in particular those related to neurotoxicity, have contributed to at least two different possible explanations of how HIV-1 initiates brain injury, the “direct injury” and the “indirect” or “bystander effect” hypothesis. These two hypothetical mechanisms are by no means mutually exclusive, and the available data suggest a role for both. However, under conditions where glial and neuronal cells are present, the indirect neurotoxicity mediated by macrophages and microglia may predominate [43,53,78,79,92,99,107,108,109].

The hypothesis that HIV proteins can directly injure neurons without any contribution of non-neuronal cells (microglia/macrophages and/or astrocytes) is supported by experiments showing that viral envelope protein gp120, Tat, and Vpr are toxic in serum free primary neuronal cultures [87,88] or in neuroblastoma cell lines [86,99,102]. The absence of non-neuronal cells permits the investigation of potential direct effects of viral proteins on neurons, but a predominantly indirect effect cannot be detected.

In mixed neuronal/glial cultures that recapitulate the cellular composition of the brain, HIV-1, gp120 and Tat appear to induce neuronal apoptotic death primarily in an indirect fashion via the release of toxins from macrophages/microglia [89,93,94,95,97,98,107,109,110]. Moreover, both the inactivation or depletion of macrophages and microglia basically abrogate neurotoxicity of HIV-1/gp120 in mixed neuronal/glial cultures [89,98,107,111] and it appears likely that stimulation of CXCR4 or CCR5 in macrophages/microglia is a prerequisite for the neurotoxicity of HIV-1 and gp120 [89,96,109].

At least some of the neurotoxins released by HIV-1 infected or gp120-stimulated macrophages/microglia stimulate an ionotropic glutamate and neurotransmitter receptor, the *N*-methyl-d-aspartate-type receptor (NMDAR) [97,112,113]. NMDAR antagonists can prevent neuronal cell death in vitro due to HIV-infected or gp120-activated macrophages [97,112]. Activation of ionotropic glutamate receptors in neurons initiates under normal physiological conditions a transient depolarization and excitation that plays an important role in neurocognitive function [114,115]. In contrast, excessive and/or extended NMDAR stimulation causes excitotoxicity via a mechanism involving a sustained elevation of intracellular Ca^2+^ concentration, a subsequent compromise of mitochondrial function and cellular energy metabolism which in turn results in the production of free radicals [43,115,116]. In case of a mild but sustained insult, as it seems to occur in HIV infection, neurons eventually undergo programmed cell death (apoptosis) which has also been observed in *post-mortem* brains of HIVE/HAD patient [71,72,89,96]. Neuronal apoptosis triggered by HIV-1 or gp120 toxicity or a direct excitotoxic insult involves neuronal Ca^2+^ overload, activation of p38 MAPK and p53, activation of cell cycle protein, caspases, release of cytochrome c and other molecules, such as apoptosis-inducing factor (AIF) from mitochondria, free radical formation, lipid release and peroxidation, and chromatin condensation [84,89,90,98,109,117,118,119,120,121]. Alterations of cellular lipid metabolism, and an increase in ceramide, sphingomyelin and hydroxynonenal as well as the activation of the unfolded protein response (UPR) have also been linked to oxidative processes and cellular distress in the neurotoxic pathways associated with HIV infection [99,122,123].

## 3. Neurotoxicity of Antiretroviral Drugs

ARV drugs are classified based on the mechanism action and currently include (i) nucleoside/nucleotide reverse transcriptase inhibitors (NRTI); (ii) non-nucleoside reverse transcriptase inhibitors (NNRTI); (iii) protease inhibitors (PI), (iv) integrase strand transfer inhibitors (INSTI); (v) a fusion inhibitor (FI); and (vi) an entry inhibitor (EI), blocking chemokine receptor CCR5. In addition, two drugs, ritonavir (RTV) and cobicistat (COBI) are used as pharmacokinetic enhancers [124] (reviewed in [125,126]). The Department of Health and Human Services (HHS) Panel on Antiretroviral Guidelines for Adults and Adolescents (a working group of the office of AIDS research) [124] keep updating the combination regimens, based on more than 25 antiretroviral drugs approved by the Food and Drug Administration (FDA) in order to guide to the right and best treatment for HIV^+^ individuals. Currently recommended cART regimen consist of two NRTIs (usually abacavir/lamivudine (ABC/3TC), tenofovir alafenamide/emtricitabine (TAF/FTC) or tenofovir disoproxil fumarate/emtricitabine (TDF/FTC)) in combination with a third active ARV drug from one of three drug classes: INSTI, NNRTI or PI with a pharmacokinetic enhancer (RTV or COBI). Other combination and/or regimen are selected based primarily on antiviral efficacy, potential adverse effects (toxicity), pill burden, drug-drug interaction, comorbid conditions and cost (see [124] on 12/1/2016). As a result of effective cART, HIV-1 infection has become a chronic condition and infected individuals have a longer, near normal life span leading to aging with HIV as a new clinical phenomenon [51,127,128,129].

The introduction of cART clearly led to a decline in the incidence of the most severe form of HAND, HAD [29,30]. However, the occurrence of cognitive impairment milder than HAD persists in HIV patients on cART [9,11,31] and recent studies observed that discontinuation of cART in virologically controlled HIV patients unexpectedly resulted in significant improvement of neurocognitive function [32,33,34,35]. Furthermore, clinical and experimental evidence is accumulating that at least some ARV compounds may themselves have neurotoxic effects [36,37,38,39,40,125,130].

Certain ARV or combinations thereof (cART) have been reported to be toxic in the periphery but also the brain, which could contribute to the development of HAND [32,36,37,38,39,40,131,132]. Several other studies have provided direct evidence that anti-retroviral drugs can exert neurotoxic effects in connection with oxidative stress, dysregulation of Ca^2+^ homeostasis and alteration of mitochondrial respiration [39,40,133,134,135,136,137,138,139].

NRTIs: These compounds have primarily been linked to peripheral neuropathy, in particular didanosine (ddI), stavudine (d4T) and zalcitabine (ddC) [140]. The main unintentional target appears to be mitochondrial polymerase γ, an enzyme required to maintain mitochondrial DNA (mtDNA) in axons and Schwann cells [141,142]. Examples of NRTIs that seem less or non-toxic in terms of peripheral neuropathy or cellular neurotoxicity are emtricitabine (FTC), lamivudine (3TC), tenofovir (TAF or TDF) and abacavir (ABC) [39]. The earliest anti-retroviral drug zidovudine (AZT) has also been linked to mitochondrial toxicity, impaired neurogenesis and damage to neuronal dendrites and presynaptic terminals [131,143,144,145]. Overall, CNS neurotoxicity of NRTIs seems limited but also compound- and cell-specific [39,125].

NNRTIs: Compounds, such as rilpivirine (TMC278) and delavirdine (DLV) are considered to be non-toxic in the CNS [125]. Etravirine (ETR) recently appeared to be clinically safe but was found to be neurotoxic in vitro [39,146]. However, Efavirenz (EFV) and nevirapine (NVP), which are FDA approved and used in NNRTI regimen [124], have been found to exert neurotoxicity [39,137,147]. EFV has also been linked in the clinical setting to deterioration of neurocognitive function [127,130,148]. One study found that EFV caused in neuron-like SHSY-5Y cells and primary rat striatal neurons a loss of ATP, depolarization and fragmentation of mitochondria and increased mitophagy and autophagy in general suggesting disturbance of energy homeostasis as a mechanism of toxicity [149,150]. EFV was also found to cause endoplasmic reticulum (ER) stress in human brain endothelial cells and in microvessels of the CNS in HIV-transgenic mice [151]. However, the latter study found that EFV inhibited autophagy by binding to a complex comprising Beclin 1, autophagy-related 14 (ATG14) and Phosphatidyl inositol 3 kinase III (PI3KIII) which is required for formation of an autophagosome. In addition, an enzyme of the cytochrome P450 (CYP) family, CYP2B6, affects the metabolism and therefore likely the concentration of EFV in the brain, which may contribute to functional impairment in the CNS as the 8-hydroxy metabolite of EFV has been found to be neurotoxic [148,152].

PIs: This group of ARV is very effective and part of most first line treatment regimen [153]. Saquinavir (SQV) and nelfinavir (NFV) were also found to be very well tolerated in HIV patients whereas ritonavir (RTV) was associated with adverse and toxic effects [154]. PI, NRTI, INSTI and the CCR5 blocker maraviroc are metabolized by enzymes of the CYP450 family. In fact, CYP450 enzymes are known to be involved in the metabolism/activation/inactivation of the majority of pharmaceutical drugs [155]. Most of the human CYP450s are primarily membrane-associated proteins located in the inner membrane of the mitochondria or in the endoplasmic reticulum of cells. Especially the subclasses CYP2 and CYP3 are involved in the metabolism of drugs and steroids, which explains the risk of drug-drug interactions in cases of polypharmacy, such as cART. However, those interactions can on occasion be harnessed for therapeutic purposes. As such RTV has been used as a booster for mono- or triple therapy [156]. Other PIs, namely amprenavir (APV), indinavir (IDV) and atazanavir (ATV) have also been implicated in neurotoxicity [157,158,159]. Moreover, a recent study demonstrated in non-human primates, rodents and in vitro neuroglial cell cultures that the PIs SQY and ATV in combination with the NRTI tenofovir (TDF) and an INSTI and RTV and SQV separately caused endoplasmic reticulum (ER) stress, activated β-site amyloid precursor protein cleaving enzyme-1 (BACE-1) and neuronal damage [160].

INSTIs: Several reports have indicated possible CNS toxicity for raltegravir (RAL) and elvitegravir (EVG) as the compounds triggered clinical psychiatric symptoms [161,162,163].

FI: The only FDA approved compound, enfuvirtide (T-20) has not displayed any conclusive evidence for neurotoxicity. Some earlier studies suggested sensory neuropathy [164,165] while others found no signs of toxicity [164,166,167].

EI: The CCR5 blocker maraviroc (MVC) is the only FDA-approved compounds in its class. There is currently no evidence of neurotoxicity, and on the contrary, several studies found evidence for a neuroprotective effect of MVC [39,92,168,169,170].

As discussed above, certain ARV and cART carry the risk of neurotoxicity. One of the major concerns using cART is the role of drug-drug interaction via various CYP450 enzymes, which could aggravate neurotoxicity and contribute to the development of HAND. Therefore the need exists for a better understanding of the mechanism of not only individual effects of ARV but their combinations in order to identify the most effective and least toxic cART for HIV-infected individuals.

## 4. Neurotoxicity of METH

METH is an addictive psychostimulant drug, and its abuse can cause a number of acute and chronic symptoms, including agitation, anxiety, paranoia, psychosis and aggression [20,22,171], a variety of cardiovascular problems [172,173], reactive microgliosis [174], and hyperthermia and convulsions [175]. Abuse of METH is also associated with behavioral symptoms, including increased engagement in high-risk activities, such as unprotected sex [20,21,22,176,177]. Neurocognitive sequelae of chronic METH abuse include deficits in attention, working memory and executive functions [28,176,178,179,180,181]. In combination, METH and HIV-1 appear to cause more neurocognitive deficits than either agent alone, but the potential mechanistic interaction of both the virus and the drug is still poorly understood [14,26,27].

The neuropathology resulting from METH abuse in humans includes a decrease of dopamine (DA) transporter (DAT) in cortex, caudate-putamen and other brain regions [182], reduction of DA and D2DA receptor in caudate-putamen [183,184], decreased density of serotonin transporter (5-HTT) in cortex [185], and abnormal glucose metabolism [186]. A pronounced interference with the nigrostriatal dopaminergic neuronal system suggests a far reaching effect on dopamine-rich fronto-striatal-thalamo-cortical circuits [187]. Moreover, a loss of gray matter in cingulate, limbic and paralimbic cortices, a significantly smaller hippocampus, and a hypertrophy of white matter and pronounced microglial activation occur as a consequence of METH use and point to pathological effects beyond the dopaminergic system [188,189].

In agreement with the findings in humans, animal studies show that METH injures presynaptic DA and 5-HT terminals [190,191], causes a decrease of tyrosine hydroxylase (TH) and tryptophan hydroxylase (TPH) [192], depletes DA and 5-HT [193], reduces DAT, 5-HTT [194,195,196] and vesicular monoamine transporter (VMAT)-2 [197]. METH also triggers neuronal cell death in cortex [198,199], striatum [198], hippocampus [200] and olfactory bulb [201]. In the striatum, METH destroys specifically gamma-amino butyric acid (GABA)-ergic neurons that express enkephalin, but not those containing substance P, neuronal nitric oxide synthase (nNOS) or cholinergic markers [202,203].

The psychostimulant effect of METH is believed to result from an elevated extracellular DA concentration in the striatum due to increased release and reduced reuptake into vesicles [179,204,205,206]. Possibly via the increase of DA, METH appears to indirectly affect glutamatergic, GABAergic and serotonergic neurotransmission. One possible link involves striatal medium spiny neurons which receive synaptic input from nigrostriatal DA-ergic and corticostriatal glutamatergic neurons [179,207].

On the other hand, in macrophages and dendritic cells, METH compromises phagocytosis and antigen processing, important processes in building and maintaining a functional and protective immune response [28,178,179,208,209,210].

Other cellular effects of METH in the brain are reflected by signs of oxidative stress, neuroinflammation and apoptosis [211,212]. Abuse of METH seems to cause excessive neuronal release of monoamine neurotransmitters, particularly DA, in the synapse, which can be toxic to nerve terminals [213,214]. As a result of DA accumulation, levels of free radicals increase inside neurons, dysfunction of the ubiquitin-proteasome system ensues, oxidative protein nitration occurs, endoplasmic reticulum stress (ERS) and expression of p53 and inflammatory cytokines is induced, and microtubules may be deacetylated [215,216,217,218]. All these processes promote neurotoxicity via damage and/or dysfunction of proteins or cellular organelles, resulting in up-regulation of autophagy, a vital homeostatic mechanism required for maintenance of healthy and functional neurons [215,216,217,218].

## 5. Neuronal Injury by HIV + cART + METH

HIV infection is frequently linked with the recreational use of drugs, such as the psychostimulant METH [20,21,22,219]. Moreover, the use of METH increases the risk of infection with HIV-1 [20,21,22]. Recent clinic-based surveys found that depending on the demographics between 16% of heterosexual men and 15% of women and up to 20% to 50% of HIV-infected men who have sex with men (MSM) reported METH use over the past 12 months [21,219,220]. Thus, the brain of many HIV patients is exposed to HIV-1, combinations of ARVs and psychostimulants, such as METH, at the same time.

Elevated viral loads have been linked to METH use in ART–receiving HIV positive individuals [23,24] and METH-users with HIV also have shown greater neuronal injury compared with patients who do not abuse the drug [25,27]. The combination of METH and HIV-1 seems to cause more neurocognitive deficits and neuropathology than either agent alone [28,219,221]. We and others have been able to recapitulate some of those combined effects in animal models [219,222,223,224,225]. However, the potential mechanistic interaction of virus and psychostimulant drug is still incompletely understood and further complicated by the multitude of cART regimen [28,145,219,221].

Therefore, our group set out to investigate in vitro the potential contribution of all these factors to neuronal injury and loss [145]. In our study, we exposed mixed neuronal-glial cerebrocortical cells to ARVs of four different pharmacological categories (NRTI: AZT, NNRTI: NVP, PI: SQV and INSTI 118-D-24) with and without METH, and in some experiments HIV-1 gp120, an established neurotoxicity inducing viral protein. The incubations lasted for 24 h and 7 days in order to assess more acute, short-term and long-term effects, respectively. Subsequently, we assessed neuronal injury using different approaches. First, the exposed cell cultures were analyzed by fluorescence microscopy using specific markers for neuronal dendrites and pre-synaptic terminals; Second, we analyzed the disturbance of neuronal ATP levels; and third, we assessed involvement of autophagy using immunofluorescence and Western blotting. We found that ARVs led to alterations of neurites and presynaptic terminals predominantly during the 7-day incubation and depending on the specific compounds and their combinations with and without METH. Similarly, specific drug combinations with and without METH or viral gp120 as well as METH and gp120 each alone, all caused a significant loss of neuronal ATP, but none of the ARV applied as a single drug. Loss of ATP was accompanied by activation of adenosine monophosphate-activated protein kinase (AMPK) and autophagy, which, however, did not suffice to restore neuronal ATP to normal levels. In contrast, enhancing autophagy with rapamycin averted the long-term drop of ATP during exposure to cART in combination with METH or gp120.

Our study yielded a number of unexpected findings pointing to the complexities of the overall effects of exposure to HIV + cART + METH [145]. The psychostimulant drug, cART and HIVgp120 each alone and in combination led to a similar loss of neuronal ATP but lacked any additive effect. Moreover, METH or HIV/gp120 or an ARV each could exert neurotoxicity in terms of compromising neuronal dendrites and presynaptic terminals or diminishing neuronal ATP or, in the case of gp120, triggering the loss of neurons themselves when applied separately, but not necessarily when combined with ARV. Certain ARV combinations did not affect neuronal dendrites or presynaptic terminal in any detectable fashion in the presence or absence of METH, yet caused significant reduction of neuronal ATP levels. Thus, neurons were able to maintain their overall dendritic and presynaptic structures despite a disruption of neuronal energy homeostasis, an event that may explain, at least in part, progressive neurological diseases, such as HAND. Strikingly, METH, which itself is toxic to neurons [28], lacked any detectable damaging effect on neurites and synapses in combination with several ARVs and even ameliorated a significant reduction of neuronal ATP in combination with a cocktail of four ARVs. However, the admixture of four therapeutic compounds and psychostimulant compromised neuronal dendrites and synapses during long-term exposure, an effect that has also been reported by others as a result of ARV treatment [39]. Moreover, diminished neuronal ATP levels resulted when four ARVs and METH were combined with HIV-1 gp120.

All those observations were in line with earlier reports that ARVs can compromise mitochondria at various levels by altering (i) mitochondrial membrane components, such as transporters; (ii) mitochondrial kinases, such as Thymidine kinase 2 (TK2) and deoxyguanosine kinase (dGK) [138]; (iii) mitochondrial bioenergetics, in particular membrane potential; and (iv) mitochondrial DNA homeostasis by inhibiting polymerase γ [134,135,136] or promote major deletions in neuronal mtDNA [133,139]. Interestingly, our findings also suggested that neuronal energy homeostasis could significantly change without causing overt structural neuronal damage or loss. The regulatory mechanism allowing for such neuronal adaptation remains to be elucidated.

Mitochondria are the cellular organelles supplying energy in the form of ATP. The lack of ATP as an energy source could be one of the factors that compromise neuronal function before cell death occurs. Neurons are known to produce energy almost exclusively through mitochondrial oxidative phosphorylation (OXPHOS). In contrast, astrocytes can stimulate glycolysis by activation of 6-phosphofructo-2 kinase (PFK2) through AMPK in order to maintain ATP levels. However, expression of PFK2 is low in neurons rendering this pathway insufficient [226,227]. Neuronal stress, such as the above discussed excitotoxicity or disturbance of the ER, may cause a disruption of mitochondrial homeostasis and consequently diminish OXPHOS. This situation is often associated with production of more reactive oxygen species (ROS) than the intracellular redox system can detoxify and precedes neurite retraction and eventual cell death [228]. Oxidatively damaged mitochondria undergo constant remodeling and turnover in neurons, a process that also demands energy [229], as functional mitochondria are transported along the microtubules to remote axonal regions [230], and conversely dysfunctional mitochondria are returned to the cell body for degradation [231]. The induction of endogenous anti-oxidant factors can apparently block the toxicity of ARV drugs, but it is not known if those protective measures are associated with normal neuronal ATP levels [40]. Maintenance of a functional population of mitochondria is critical for postmitotic neurons, and is regulated mainly by autophagy of the damaged organelles, a process called mitophagy [232,233]. This clearance usually contributes to maintenance or the re-establishment of baseline cellular ATP levels [234,235] and is mainly controlled by AMPK and mTOR signaling pathways [236]. Since ARV drugs, METH and HIV-1 or its fragments, including gp120, all generate in neurons oxidative stress and loss of ATP, activation of autophagy seems to provide a suitable protective mechanism [40,145,216]. The importance of the cellular clearance process is supported by several studies showing that impaired autophagy in neurons contributes to the development of neurodegeneration [237,238].

**Figure 1:**
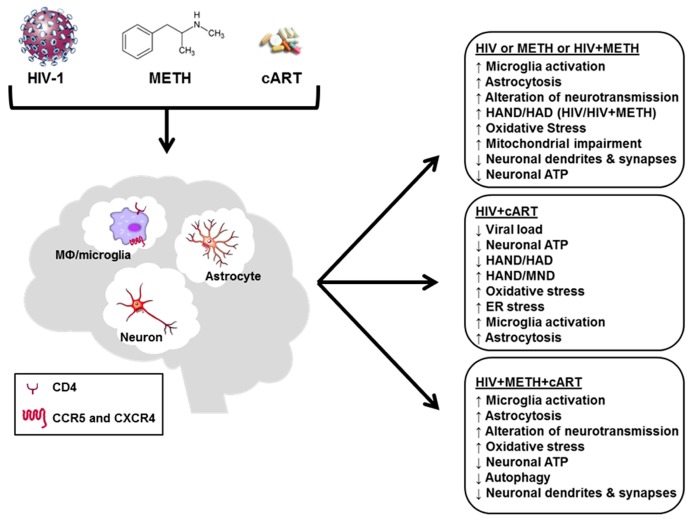
Treatment of HIV-1 infection with cART greatly reduces viral loads in periphery and the central nervous system (CNS), the incidence of HIV-associated dementia (HAD) and acquired immunodeficiency syndrome (AIDS)-related deaths. HIV-1 or its components, methamphetamine (METH) and some antiretrovirals used in combined antiretroviral therapy (cART) can induce oxidative and endoplasmic retriculum (ER) stress, compromise autophagy and neuronal energy homeostasis and trigger functional as well as structural neuronal injury. However, even without overt structural damage, diminished adenosine triphosphate (ATP) levels indicate that neurons may lose, at least in part, the energy reserve that is vital to maintaining their normal membrane potential and physiological functions, such as homeostatic regulation of neurotransmission. A compromised neuronal energy homeostasis may explain why cART permits the occurrence of HIV-associated mild neurocognitive disorders (MND). Interestingly, neuronal energy levels can apparently drop significantly during exposure to some drugs or combinations thereof without necessarily destructing neuronal dendrites and pre-synaptic terminals, suggesting that a restoration of full neuronal function may be possible.

Since neurons induced autophagy in the presence of in HIV gp120 or cART or METH and combination thereof but failed to recover homeostatic levels of ATP, we employed rapamycin to block mTOR, which is an inhibitor of autophagy [145,239]. In the presence of cART, METH and gp120, rapamycin helped to restore neuronal ATP to control levels. Thus, neurons were in principle able to restore ATP levels by autophagy but failed to do so when challenged separately with gp120 or the ARV cocktail or in the absence of rapamycin for reasons that need to be further explored. Strikingly, rapamycin also preserved normal neuronal ATP levels over 7 days in the presence of the combination of cART and gp120 without METH or the combination of METH and gp120 without cART. This outcome might be explained if cART and gp120 engaged different disturbance mechanisms to push neuronal ATP homeostasis off balance. Those mechanisms could however neutralize each other when ARVs and gp120 were combined or could be modified by the presence of METH. The loss of ATP associated with METH exposure was significantly ameliorated by rapamycin but not completely prevented [145]. A possible explanation may be related to the observation that autophagy can cause neurite degeneration in the presence of METH [216]. Thus, autophagy may not always be protective and perhaps be associated with a risk of damaging neuronal structure. Figure 1 provides a schematic summary of the various aspects of the brain’s exposure to HIV + METH + cART.

While we are beginning to untangle the combined effects of HIV + cART + METH by characterizing the associated cellular events, more work lies ahead. Studying the combination of HIV and_cART with other abused drugs, such as heroin and cocaine, seems equally important as these substances may involve other pathways and affect HIV neuropathogenesis in different ways than METH [15,16,17,18,240]. Moreover, we largely focused on neuronal injury, but METH as well as other abused drugs also affect the immune system and HIV infection itself by compromising for example the interferon response [241,242,243,244]. Opioids, cocaine and METH are highly addictive and all seem to be able to influence gene expression in neural and immune cells via mechanisms that involve short, con-coding, regulatory microRNAs and compromise control of HIV infection as well as normal neuronal function [19,245]. Moreover, accumulating evidence suggests a critical role for exosomes, globular, membranous extracellular nanovesicles in HIV infection, HAND and also drug abuse-related neurodegenerative diseases [246,247,248,249]. A detailed discussion of the additional abused substances, microRNAs and exosomes is beyond the scope of this review, but those areas of research are poised to significantly contribute to our understanding of the combined effects of HIV + cART + abused drugs on the brain and may help to develop future, improved therapies for addiction and HAND.

## 6. Conclusions

The introduction of cART has transformed HIV-1 infection into a treatable, yet chronic disease that remains associated with a high probability for development of neurocognitive impairment, now termed HAND. Accumulating evidence points to the possibility that besides HIV-1 itself long-term exposure to cART can also contribute to the occurrence of HAND. The fact that HIV-1 infection is frequently associated with the abuse or recreational psychostimulant drugs further complicates the situation. Several lines of evidence indicate that similar to HIV-1 or its components and METH, some ARV used in cART can also induce oxidative and ER stress, compromise neuronal energy homeostasis and trigger structural neuronal injury. However, even without overt structural damage, diminished ATP levels indicate that neurons may lose, at least in part, the energy reserve that is vital to maintaining their normal membrane potential and physiological function. Interestingly, neuronal energy levels can apparently drop significantly during exposure to some drugs or combinations thereof without necessarily destructing neuronal dendrites and pre-synaptic terminals, suggesting that a restoration of full neuronal function may be possible. Altogether, the available data suggest that the overall beneficial effect of cART against HIV infection can be accompanied by a discernable level of neurotoxicity that may be modified, but in the long-term primarily aggravated by the use of psychostimulant drugs, such as methamphetamine.

## References

[1] Fauci A.S. (1999). The aids epidemic—Considerations for the 21st century. N. Engl. J. Med..

[2] Piot P., Bartos M., Ghys P.D., Walker N., Schwartlander B. (2001). The global impact of HIV/AIDS. Nature.

[3] Fact Sheet November 2016. http://www.unaids.org/en/resources/fact-sheet.

[4] The Joint United Nations Programme on HIV/AIDS (UNAIDS) Unaids Report on the Global Aids Epidemic 2013. http://www.unaids.org/sites/default/files/media_asset/UNAIDS_Global_Report_2013_en_1.pdf.

[5] The Joint United Nations Programme on HIV/AIDS (UNAIDS) Global Aids Response Progress Reporting 2015. http://www.unaids.org/sites/default/files/media_asset/JC2702_GARPR2015guidelines_en.pdf.

[6] Centers for Disease Control and Prevention. https://www.cdc.gov/hiv/.

[7] Antinori A., Arendt G., Becker J.T., Brew B.J., Byrd D.A., Cherner M., Clifford D.B., Cinque P., Epstein L.G., Goodkin K. (2007). Updated research nosology for HIV-associated neurocognitive disorders. Neurology.

[8] Kaul M. (2008). Hiv‘s double strike at the brain: Neuronal toxicity and compromised neurogenesis. Front. Biosci..

[9] McArthur J.C., Steiner J., Sacktor N., Nath A. (2010). Human immunodeficiency virus-associated neurocognitive disorders: Mind the gap. Ann. Neurol..

[10] Heaton R.K., Clifford D.B., Franklin D.R., Woods S.P., Ake C., Vaida F., Ellis R.J., Letendre S.L., Marcotte T.D., Atkinson J.H. (2010). HIV-associated neurocognitive disorders persist in the era of potent antiretroviral therapy: Charter study. Neurology.

[11] Saylor D., Dickens A.M., Sacktor N., Haughey N., Slusher B., Pletnikov M., Mankowski J.L., Brown A., Volsky D.J., McArthur J.C. (2016). HIV-associated neurocognitive disorder—Pathogenesis and prospects for treatment. Nat. Rev. Neurol..

[12] Drugs of Abuse. https://www.drugabuse.gov/drugs-abuse.

[13] Peterson P.K., Gekker G., Schut R., Hu S., Balfour H.H., Chao C.C. (1993). Enhancement of HIV-1 replication by opiates and cocaine: The cytokine connection. Adv. Exp. Med. Biol..

[14] Carey C.L., Woods S.P., Rippeth J.D., Gonzalez R., Heaton R.K., Grant I. (2006). Additive deleterious effects of methamphetamine dependence and immunosuppression on neuropsychological functioning in HIV infection. AIDS Behav..

[15] Byrd D.A., Fellows R.P., Morgello S., Franklin D., Heaton R.K., Deutsch R., Atkinson J.H., Clifford D.B., Collier A.C., Marra C.M. (2011). Neurocognitive impact of substance use in HIV infection. J. Acquir. Immune Defic. Syndr..

[16] Buch S., Yao H., Guo M., Mori T., Mathias-Costa B., Singh V., Seth P., Wang J., Su T.P. (2012). Cocaine and HIV-1 interplay in CNS: Cellular and molecular mechanisms. Curr. HIV Res..

[17] Silverstein P.S., Shah A., Weemhoff J., Kumar S., Singh D.P., Kumar A. (2012). HIV-1 gp120 and drugs of abuse: Interactions in the central nervous system. Curr. HIV Res..

[18] Chang S.L., Connaghan K.P., Wei Y., Li M.D. (2014). Neurohiv and use of addictive substances. Int. Rev. Neurobiol..

[19] Pilakka-Kanthikeel S., Nair M.P. (2015). Interaction of drugs of abuse and microrna with HIV: A brief review. Front. Microbiol..

[20] Kapadia F., Vlahov D., Donahoe R.M., Friedland G. (2005). The role of substance abuse in HIV disease progression: Reconciling differences from laboratory and epidemiologic investigations. Clin. Infect. Dis..

[21] Mitchell S.J., Morris S.R., Kent C.K., Stansell J., Klausner J.D. (2006). Methamphetamine use and sexual activity among HIV-infected patients in care—San Francisco, 2004. Aids Patient Care STDs.

[22] Urbina A., Jones K. (2004). Crystal methamphetamine, its analogues, and HIV infection: Medical and psychiatric aspects of a new epidemic. Clin. Infect. Dis..

[23] Hinkin C.H., Barclay T.R., Castellon S.A., Levine A.J., Durvasula R.S., Marion S.D., Myers H.F., Longshore D. (2007). Drug use and medication adherence among HIV-1 infected individuals. AIDS Behav..

[24] Ellis R.J., Childers M.E., Cherner M., Lazzaretto D., Letendre S., Grant I. (2003). Increased human immunodeficiency virus loads in active methamphetamine users are explained by reduced effectiveness of antiretroviral therapy. J. Infect. Dis..

[25] Langford T.D., Letendre S.L., Larrea G.J., Masliah E. (2003). Changing patterns in the neuropathogenesis of HIV during the haart era. Brain Pathol..

[26] Rippeth J.D., Heaton R.K., Carey C.L., Marcotte T.D., Moore D.J., Gonzalez R., Wolfson T., Grant I. (2004). Methamphetamine dependence increases risk of neuropsychological impairment in HIV infected persons. J. Int. Neuropsychol. Soc..

[27] Chana G., Everall I.P., Crews L., Langford D., Adame A., Grant I., Cherner M., Lazzaretto D., Heaton R., Ellis R. (2006). Cognitive deficits and degeneration of interneurons in HIV+ methamphetamine users. Neurology.

[28] Cadet J.L., Krasnova I.N. (2007). Interactions of HIV and methamphetamine: Cellular and molecular mechanisms of toxicity potentiation. Neurotox. Res..

[29] Eisfeld C., Reichelt D., Evers S., Husstedt I. (2013). Csf penetration by antiretroviral drugs. CNS Drugs.

[30] Heaton R.K., Franklin D.R., Deutsch R., Letendre S., Ellis R.J., Casaletto K., Marquine M.J., Woods S.P., Vaida F., Atkinson J.H. (2015). Neurocognitive change in the era of HIV combination antiretroviral therapy: The longitudinal charter study. Clin. Infect. Dis..

[31] Heaton R.K., Franklin D.R., Ellis R.J., McCutchan J.A., Letendre S.L., Leblanc S., Corkran S.H., Duarte N.A., Clifford D.B., Woods S.P. (2011). HIV-associated neurocognitive disorders before and during the era of combination antiretroviral therapy: Differences in rates, nature, and predictors. J. Neurovirol..

[32] Evans S.R., Ellis R.J., Chen H., Yeh T.M., Lee A.J., Schifitto G., Wu K., Bosch R.J., McArthur J.C., Simpson D.M. (2011). Peripheral neuropathy in HIV: Prevalence and risk factors. AIDS.

[33] Evans S.R., Lee A.J., Ellis R.J., Chen H., Wu K., Bosch R.J., Clifford D.B. (2012). HIV peripheral neuropathy progression: Protection with glucose-lowering drugs?. J. Neurovirol..

[34] Robertson K.R., Su Z., Margolis D.M., Krambrink A., Havlir D.V., Evans S., Skiest D.J. (2010). Neurocognitive effects of treatment interruption in stable HIV-positive patients in an observational cohort. Neurology.

[35] Underwood J., Robertson K.R., Winston A. (2014). Could antiretroviral neurotoxicity play a role in the pathogenesis of cognitive impairment in treated HIV disease?. AIDS.

[36] Carr A. (2003). Toxicity of antiretroviral therapy and implications for drug development. Nat. Rev. Drug Discov..

[37] Keswani S.C., Chander B., Hasan C., Griffin J.W., McArthur J.C., Hoke A. (2003). Fk506 is neuroprotective in a model of antiretroviral toxic neuropathy. Ann. Neurol..

[38] Lewin S.R., Hoy J., Crowe S.M., McDonald C.F. (1995). The role of bronchoscopy in the diagnosis and treatment of pulmonary disease in HIV-infected patients. Aust. N. Z. J. Med..

[39] Robertson K., Liner J., Meeker R.B. (2012). Antiretroviral neurotoxicity. J. Neurovirol..

[40] Akay C., Cooper M., Odeleye A., Jensen B.K., White M.G., Vassoler F., Gannon P.J., Mankowski J., Dorsey J.L., Buch A.M. (2014). Antiretroviral drugs induce oxidative stress and neuronal damage in the central nervous system. J. Neurovirol..

[41] Navia B.A., Jordan B.D., Price R.W. (1986). The aids dementia complex: I. Clinical features. Ann. Neurol..

[42] Price R.W., Brew B., Sidtis J., Rosenblum M., Scheck A.C., Cleary P. (1988). The brain in aids: Central nervous system HIV-1 infection and aids dementia complex. Science.

[43] Kaul M., Garden G.A., Lipton S.A. (2001). Pathways to neuronal injury and apoptosis in HIV-associated dementia. Nature.

[44] Sacktor N., Lyles R.H., Skolasky R., Kleeberger C., Selnes O.A., Miller E.N., Becker J.T., Cohen B., McArthur J.C. (2001). HIV-associated neurologic disease incidence changes: Multicenter aids cohort study, 1990–1998. Neurology.

[45] Ellis R.J., Deutsch R., Heaton R.K., Marcotte T.D., McCutchan J.A., Nelson J.A., Abramson I., Thal L.J., Atkinson J.H., Wallace M.R. (1997). Neurocognitive impairment is an independent risk factor for death in hiv infection. San Diego HIV neurobehavioral research center group. Arch. Neurol..

[46] Cunningham P.H., Smith D.G., Satchell C., Cooper D.A., Brew B. (2000). Evidence for independent development of resistance to HIV-1 reverse transcriptase inhibitors in the cerebrospinal fluid. AIDS.

[47] Cysique L.A., Maruff P., Brew B.J. (2006). Variable benefit in neuropsychological function in HIV-infected haart-treated patients. Neurology.

[48] Giancola M.L., Lorenzini P., Balestra P., Larussa D., Baldini F., Corpolongo A., Narciso P., Bellagamba R., Tozzi V., Antinori A. (2006). Neuroactive antiretroviral drugs do not influence neurocognitive performance in less advanced hiv-infected patients responding to highly active antiretroviral therapy. J. Acquir. Immune Defic. Syndr..

[49] Nath A., Sacktor N. (2006). Influence of highly active antiretroviral therapy on persistence of HIV in the central nervous system. Curr. Opin. Neurol..

[50] McArthur J.C., Haughey N., Gartner S., Conant K., Pardo C., Nath A., Sacktor N. (2003). Human immunodeficiency virus-associated dementia: An evolving disease. J. Neurovirol..

[51] Brew B.J., Crowe S.M., Landay A., Cysique L.A., Guillemin G. (2009). Neurodegeneration and ageing in the haart era. J. Neuroimmune Pharmacol..

[52] Welch K., Morse A. (2002). The clinical profile of end-stage aids in the era of highly active antiretroviral therapy. AIDS Patient Care STDs.

[53] Kaul M., Zheng J., Okamoto S., Gendelman H.E., Lipton S.A. (2005). HIV-1 infection and aids: Consequences for the central nervous system. Cell Death Differ..

[54] Kramer-Hammerle S., Rothenaigner I., Wolff H., Bell J.E., Brack-Werner R. (2005). Cells of the central nervous system as targets and reservoirs of the human immunodeficiency virus. Virus Res..

[55] Lipton S.A. (1997). Treating aids dementia [letter; comment]. Science.

[56] Jones G., Power C. (2006). Regulation of neural cell survival by HIV-1 infection. Neurobiol. Dis..

[57] Petito C.K., Cho E.S., Lemann W., Navia B.A., Price R.W. (1986). Neuropathology of acquired immunodeficiency syndrome (AIDS): An autopsy review. J. Neuropathol. Exp. Neurol..

[58] Masliah E., Heaton R.K., Marcotte T.D., Ellis R.J., Wiley C.A., Mallory M., Achim C.L., McCutchan J.A., Nelson J.A., Atkinson J.H. (1997). Dendritic injury is a pathological substrate for human immunodeficiency virus-related cognitive disorders. Hnrc group. The HIV neurobehavioral research center. Ann. Neurol..

[59] Glass J.D., Fedor H., Wesselingh S.L., McArthur J.C. (1995). Immunocytochemical quantitation of human immunodeficiency virus in the brain: Correlations with dementia. Ann. Neurol..

[60] Achim C.L., Wang R., Miners D.K., Wiley C.A. (1994). Brain viral burden in HIV infection. J. Neuropathol. Exp. Neurol..

[61] Wiley C.A., Masliah E., Achim C.L. (1994). Measurement of cns HIV burden and its association with neurologic damage. Adv. Neuroimmunol..

[62] Wesselingh S.L., Takahashi K., Glass J.D., McArthur J.C., Griffin J.W., Griffin D.E. (1997). Cellular localization of tumor necrosis factor mrna in neurological tissue from HIV-infected patients by combined reverse transcriptase/polymerase chain reaction in situ hybridization and immunohistochemistry. J. Neuroimmunol..

[63] Heyes M.P., Brew B.J., Martin A., Price R.W., Salazar A.M., Sidtis J.J., Yergey J.A., Mouradian M.M., Sadler A.E., Keilp J. (1991). Quinolinic acid in cerebrospinal fluid and serum in HIV-1 infection: Relationship to clinical and neurological status. Ann. Neurol..

[64] Shiramizu B., Gartner S., Williams A., Shikuma C., Ratto-Kim S., Watters M., Aguon J., Valcour V. (2005). Circulating proviral HIV DNA and HIV-associated dementia. AIDS.

[65] Shiramizu B., Ratto-Kim S., Sithinamsuwan P., Nidhinandana S., Thitivichianlert S., Watt G., Desouza M., Chuenchitra T., Sukwit S., Chitpatima S. (2006). HIV DNA and dementia in treatment-naive HIV-1-infected individuals in bangkok, thailand. Int. J. Med. Sci..

[66] Ketzler S., Weis S., Haug H., Budka H. (1990). Loss of neurons in the frontal cortex in aids brains. Acta Neuropathol..

[67] Everall I.P., Luthert P.J., Lantos P.L. (1991). Neuronal loss in the frontal cortex in HIV infection. Lancet.

[68] Reyes M.G., Faraldi F., Senseng C.S., Flowers C., Fariello R. (1991). Nigral degeneration in acquired immune deficiency syndrome (AIDS). Acta Neuropathol..

[69] Graus F., Ribalta T., Abos J., Alom J., Cruz-Sanchez F., Mallolas J., Miro J.M., Cardesa A., Tolosa E. (1990). Subacute cerebellar syndrome as the first manifestation of aids dementia complex. Acta Neurol. Scand..

[70] Everall I., Luthert P., Lantos P. (1993). A review of neuronal damage in human immunodeficiency virus infection: Its assessment, possible mechanism and relationship to dementia. J. Neuropathol. Exp. Neurol..

[71] Adle-Biassette H., Chrétien F., Wingertsmann L., Héry C., Ereau T., Scaravilli F., Tardieu M., Gray F. (1999). Neuronal apoptosis does not correlate with dementia in HIV infection but is related to microglial activation and axonal damage. Neuropathol. Appl. Neurobiol..

[72] Petito C.K., Roberts B. (1995). Evidence of apoptotic cell death in HIV encephalitis. Am. J. Pathol..

[73] Rostasy K., Monti L., Yiannoutsos C., Wu J., Bell J., Hedreen J., Navia B.A. (2000). Nfkappab activation, TNF-alpha expression, and apoptosis in the aids-dementia-complex. J. Neurovirol..

[74] Anthony I.C., Ramage S.N., Carnie F.W., Simmonds P., Bell J.E. (2005). Influence of haart on HIV-related cns disease and neuroinflammation. J. Neuropathol. Exp. Neurol..

[75] Everall I.P., Hansen L.A., Masliah E. (2005). The shifting patterns of HIV encephalitis neuropathology. Neurotox. Res..

[76] Green D.A., Masliah E., Vinters H.V., Beizai P., Moore D.J., Achim C.L. (2005). Brain deposition of beta-amyloid is a common pathologic feature in HIV positive patients. AIDS.

[77] Ho D.D., Rota T.R., Schooley R.T., Kaplan J.C., Allan J.D., Groopman J.E., Resnick L., Felsenstein D., Andrews C.A., Hirsch M.S. (1985). Isolation of HTLV-III from cerebrospinal fluid and neural tissues of patients with neurologic syndromes related to the acquired immunodeficiency syndrome. N. Engl. J. Med..

[78] Gartner S. (2000). HIV infection and dementia. Science.

[79] Gonzalez-Scarano F., Martin-Garcia J. (2005). The neuropathogenesis of AIDS. Nat. Rev. Immunol..

[80] Koenig S., Gendelman H.E., Orenstein J.M., Dal Canto M.C., Pezeshkpour G.H., Yungbluth M., Janotta F., Aksamit A., Martin M.A., Fauci A.S. (1986). Detection of aids virus in macrophages in brain tissue from aids patients with encephalopathy. Science.

[81] Dragic T., Litwin V., Allaway G.P., Martin S.R., Huang Y., Nagashima K.A., Cayanan C., Maddon P.J., Koup R.A., Moore J.P. (1996). HIV-1 entry into CD4+ cells is mediated by the chemokine receptor CC-CKR-5. Nature.

[82] Oberlin E., Amara A., Bachelerie F., Bessia C., Virelizier J.L., Arenzana-Seisdedos F., Schwartz O., Heard J.M., Clark-Lewis I., Legler D.F. (1996). The CXC chemokine SDF-1 is the ligand for LESTR/fusin and prevents infection by T-cell-line-adapted HIV-1. Nature.

[83] He J., Chen Y., Farzan M., Choe H., Ohagen A., Gartner S., Busciglio J., Yang X., Hofmann W., Newman W. (1997). CCR3 and CCR5 are co-receptors for HIV-1 infection of microglia. Nature.

[84] Asensio V.C., Campbell I.L. (1999). Chemokines in the CNS: Plurifunctional mediators in diverse states. Trends Neurosci..

[85] Miller R.J., Meucci O. (1999). Aids and the brain: Is there a chemokine connection?. Trends Neurosci..

[86] Hesselgesser J., Taub D., Baskar P., Greenberg M., Hoxie J., Kolson D.L., Horuk R. (1998). Neuronal apoptosis induced by HIV-1 gp120 and the chemokine SDF-1 alpha is mediated by the chemokine receptor CXCR4. Curr. Biol..

[87] Meucci O., Fatatis A., Simen A.A., Bushell T.J., Gray P.W., Miller R.J. (1998). Chemokines regulate hippocampal neuronal signaling and gp120 neurotoxicity. Proc. Natl. Acad. Sci. USA.

[88] Meucci O., Fatatis A., Simen A.A., Miller R.J. (2000). Expression of CX3CR1 chemokine receptors on neurons and their role in neuronal survival. Proc. Natl. Acad. Sci. USA.

[89] Kaul M., Lipton S.A. (1999). Chemokines and activated macrophages in HIV gp120-induced neuronal apoptosis. Proc. Natl. Acad. Sci. USA.

[90] Kaul M., Ma Q., Medders K.E., Desai M.K., Lipton S.A. (2007). HIV-1 coreceptors CCR5 and CXCR4 both mediate neuronal cell death but CCR5 paradoxically can also contribute to protection. Cell Death Differ..

[91] Zheng J., Thylin M.R., Ghorpade A., Xiong H., Persidsky Y., Cotter R., Niemann D., Che M., Zeng Y.C., Gelbard H.A. (1999). Intracellular CXCR4 signaling, neuronal apoptosis and neuropathogenic mechanisms of HIV-1-associated dementia. J. Neuroimmunol..

[92] Maung R., Hoefer M.M., Sanchez A.B., Sejbuk N.E., Medders K.E., Desai M.K., Catalan I.C., Dowling C.C., de Rozieres C.M., Garden G.A. (2014). CCR5 knockout prevents neuronal injury and behavioral impairment induced in a transgenic mouse model by a CXCR4-using HIV-1 glycoprotein 120. J. Immunol..

[93] Brenneman D.E., Westbrook G.L., Fitzgerald S.P., Ennist D.L., Elkins K.L., Ruff M.R., Pert C.B. (1988). Neuronal cell killing by the envelope protein of HIV and its prevention by vasoactive intestinal peptide. Nature.

[94] Iskander S., Walsh K.A., Hammond R.R. (2004). Human CNS cultures exposed to HIV-1 gp120 reproduce dendritic injuries of HIV-1-associated dementia. J. Neuroinflamm..

[95] Walsh K.A., Megyesi J.F., Wilson J.X., Crukley J., Laubach V.E., Hammond R.R. (2004). Antioxidant protection from HIV-1 gp120-induced neuroglial toxicity. J. Neuroinflamm..

[96] Ohagen A., Ghosh S., He J., Huang K., Chen Y., Yuan M., Osathanondh R., Gartner S., Shi B., Shaw G. (1999). Apoptosis induced by infection of primary brain cultures with diverse human immunodeficiency virus type 1 isolates: Evidence for a role of the envelope. J. Virol..

[97] Chen W., Sulcove J., Frank I., Jaffer S., Ozdener H., Kolson D.L. (2002). Development of a human neuronal cell model for human immunodeficiency virus (HIV)-infected macrophage-induced neurotoxicity: Apoptosis induced by HIV type 1 primary isolates and evidence for involvement of the Bcl-2/Bcl-xl-sensitive intrinsic apoptosis pathway. J. Virol..

[98] Garden G.A., Guo W., Jayadev S., Tun C., Balcaitis S., Choi J., Montine T.J., Moller T., Morrison R.S. (2004). HIV associated neurodegeneration requires p53 in neurons and microglia. FASEB J..

[99] Mattson M.P., Haughey N.J., Nath A. (2005). Cell death in HIV dementia. Cell Death Differ..

[100] New D.R., Ma M., Epstein L.G., Nath A., Gelbard H.A. (1997). Human immunodeficiency virus type 1 tat protein induces death by apoptosis in primary human neuron cultures. J. Neurovirol..

[101] Adamson D.C., Wildemann B., Sasaki M., Glass J.D., McArthur J.C., Christov V.I., Dawson T.M., Dawson V.L. (1996). Immunologic no synthase: Elevation in severe aids dementia and induction by HIV-1 gp41. Science.

[102] Piller S.C., Jans P., Gage P.W., Jans D.A. (1998). Extracellular HIV-1 virus protein r causes a large inward current and cell death in cultured hippocampal neurons: Implications for aids pathology. Proc. Natl. Acad. Sci. USA.

[103] Koedel U., Kohleisen B., Sporer B., Lahrtz F., Ovod V., Fontana A., Erfle V., Pfister H.W. (1999). HIV type 1 nef protein is a viral factor for leukocyte recruitment into the central nervous system. J. Immunol..

[104] Ellis R., Langford D., Masliah E. (2007). HIV and antiretroviral therapy in the brain: Neuronal injury and repair. Nat. Rev. Neurosci..

[105] Krathwohl M.D., Kaiser J.L. (2004). HIV-1 promotes quiescence in human neural progenitor cells. J. Infect. Dis..

[106] Okamoto S., Kang Y.J., Brechtel C.W., Siviglia E., Russo R., Clemente A., Harrop A., McKercher S., Kaul M., Lipton S.A. (2007). HIV/gp120 decreases adult neural progenitor cell proliferation via checkpoint kinase-mediated cell-cycle withdrawal and G1 arrest. Cell Stem Cell.

[107] Giulian D., Vaca K., Noonan C.A. (1990). Secretion of neurotoxins by mononuclear phagocytes infected with HIV-1. Science.

[108] Genis P., Jett M., Bernton E.W., Boyle T., Gelbard H.A., Dzenko K., Keane R.W., Resnick L., Mizrachi Y., Volsky D.J. (1992). Cytokines and arachidonic metabolites produced during human immunodeficiency virus (HIV)-infected macrophage-astroglia interactions: Implications for the neuropathogenesis of HIV disease. J. Exp. Med..

[109] Medders K.E., Sejbuk N.E., Maung R., Desai M.K., Kaul M. (2010). Activation of p38 mapk is required in monocytic and neuronal cells for HIV glycoprotein 120-induced neurotoxicity. J. Immunol..

[110] Sui Z., Fan S., Sniderhan L., Reisinger E., Litzburg A., Schifitto G., Gelbard H.A., Dewhurst S., Maggirwar S.B. (2006). Inhibition of mixed lineage kinase 3 prevents HIV-1 tat-mediated neurotoxicity and monocyte activation. J. Immunol..

[111] Giulian D., Wendt E., Vaca K., Noonan C.A. (1993). The envelope glycoprotein of human immunodeficiency virus type 1 stimulates release of neurotoxins from monocytes. Proc. Natl. Acad. Sci. USA.

[112] Dreyer E.B., Kaiser P.K., Offermann J.T., Lipton S.A. (1990). HIV-1 coat protein neurotoxicity prevented by calcium channel antagonists. Science.

[113] O’Donnell L.A., Agrawal A., Jordan-Sciutto K.L., Dichter M.A., Lynch D.R., Kolson D.L. (2006). Human immunodeficiency virus (HIV)-induced neurotoxicity: Roles for the nmda receptor subtypes. J. Neurosci..

[114] Olney J.W., Labruyere J., Wang G., Wozniak D.F., Price M.T., Sesma M.A. (1991). Nmda antagonist neurotoxicity: Mechanism and prevention. Science.

[115] Doble A. (1999). The role of excitotoxicity in neurodegenerative disease: Implications for therapy. Pharmacol. Ther..

[116] Olney J.W. (1969). Brain lesions, obesity, and other disturbances in mice treated with monosodium glutamate. Science.

[117] Tenneti L., D’Emilia D.M., Troy C.M., Lipton S.A. (1998). Role of caspases in *N*-methyl-d-aspartate-induced apoptosis in cerebrocortical neurons. J. Neurochem..

[118] Garden G.A., Budd S.L., Tsai E., Hanson L., Kaul M., D’Emilia D.M., Friedlander R.M., Yuan J., Masliah E., Lipton S.A. (2002). Caspase cascades in human immunodeficiency virus-associated neurodegeneration. J. Neurosci..

[119] Jordan-Sciutto K.L., Wang G., Murphey-Corb M., Wiley C.A. (2002). Cell cycle proteins exhibit altered expression patterns in lentiviral-associated encephalitis. J. Neurosci..

[120] Jana A., Pahan K. (2004). Human immunodeficiency virus type 1 gp120 induces apoptosis in human primary neurons through redox-regulated activation of neutral sphingomyelinase. J. Neurosci..

[121] Haughey N.J., Mattson M.P. (2002). Calcium dysregulation and neuronal apoptosis by the HIV-1 proteins tat and gp120. J. Acquir. Immune Defic. Syndr..

[122] Haughey N.J., Cutler R.G., Tamara A., McArthur J.C., Vargas D.L., Pardo C.A., Turchan J., Nath A., Mattson M.P. (2004). Perturbation of sphingolipid metabolism and ceramide production in HIV-dementia. Ann. Neurol..

[123] Lindl K.A., Akay C., Wang Y., White M.G., Jordan-Sciutto K.L. (2007). Expression of the endoplasmic reticulum stress response marker, BiP, in the central nervous system of HIV-positive individuals. Neuropathol. Appl. Neurobiol..

[124] Federally Approved HIV/AIDS Medical Practice Guidelines. https://aidsinfo.nih.gov/guidelines.

[125] Shah A., Gangwani M.R., Chaudhari N.S., Glazyrin A., Bhat H.K., Kumar A. (2016). Neurotoxicity in the post-haart era: Caution for the antiretroviral therapeutics. Neurotox. Res..

[126] Badowski M.E., Perez S.E., Biagi M., Littler J.A. (2016). New antiretroviral treatment for HIV. Infect. Dis. Ther..

[127] Ciccarelli N., Fabbiani M., Di G.S., Fanti I., Baldonero E., Bracciale L., Tamburrini E., Cauda R., De L.A., Silveri M.C. (2011). Efavirenz associated with cognitive disorders in otherwise asymptomatic HIV-infected patients. Neurology.

[128] Canizares S., Cherner M., Ellis R.J. (2014). HIV and aging: Effects on the central nervous system. Semin. Neurol..

[129] DeVaughn S., Muller-Oehring E.M., Markey B., Bronte-Stewart H.M., Schulte T. (2015). Aging with HIV-1 infection: Motor functions, cognition, and attention—A comparison with parkinson’s disease. Neuropsychol. Rev..

[130] Ma Q., Vaida F., Wong J., Sanders C.A., Kao Y.T., Croteau D., Clifford D.B., Collier A.C., Gelman B.B., Marra C.M. (2016). Long-term efavirenz use is associated with worse neurocognitive functioning in HIV-infected patients. J. Neurovirol..

[131] Lewis W., Dalakas M.C. (1995). Mitochondrial toxicity of antiviral drugs. Nat. Med..

[132] Van Dyke R.B., Wang L., Williams P.L., Pediatric AIDS Clinical Trials Group 219C Team (2008). Toxicities associated with dual nucleoside reverse-transcriptase inhibitor regimens in hiv-infected children. J. Infect. Dis..

[133] Apostolova N., Blas-Garcia A., Esplugues J.V. (2011). Mitochondrial toxicity in haart: An overview of in vitro evidence. Curr. Pharm. Des..

[134] Arnaudo E., Dalakas M., Shanske S., Moraes C.T., DiMauro S., Schon E.A. (1991). Depletion of muscle mitochondrial DNA in aids patients with zidovudine-induced myopathy. Lancet.

[135] Cherry C.L., Gahan M.E., McArthur J.C., Lewin S.R., Hoy J.F., Wesselingh S.L. (2002). Exposure to dideoxynucleosides is reflected in lowered mitochondrial DNA in subcutaneous fat. J. Acquir. Immune Defic. Syndr..

[136] Cote H.C., Brumme Z.L., Craib K.J., Alexander C.S., Wynhoven B., Ting L., Wong H., Harris M., Harrigan P.R., O’Shaughnessy M.V. (2002). Changes in mitochondrial DNA as a marker of nucleoside toxicity in HIV-infected patients. N. Engl. J. Med..

[137] Funes H.A., Apostolova N., Alegre F., Blas-Garcia A., Alvarez A., Marti-Cabrera M., Esplugues J.V. (2014). Neuronal bioenergetics and acute mitochondrial dysfunction: A clue to understanding the central nervous system side effects of efavirenz. J. Infect. Dis..

[138] Sun R., Eriksson S., Wang L. (2014). Down-regulation of mitochondrial thymidine kinase 2 and deoxyguanosine kinase by didanosine: Implication for mitochondrial toxicities of anti-HIV nucleoside analogs. Biochem. Biophys. Res. Commun..

[139] Zhang Y., Song F., Gao Z., Ding W., Qiao L., Yang S., Chen X., Jin R., Chen D. (2014). Long-term exposure of mice to nucleoside analogues disrupts mitochondrial DNA maintenance in cortical neurons. PLoS ONE.

[140] Dragovic G., Jevtovic D. (2003). Nucleoside reverse transcriptase inhibitor usage and the incidence of peripheral neuropathy in HIV/AIDS patients. Antivir. Chem. Chemother..

[141] Dalakas M.C. (2001). Peripheral neuropathy and antiretroviral drugs. J. Peripher. Nerv. Syst..

[142] Venhoff N., Lebrecht D., Deveaud C., Beauvoit B., Bonnet J., Muller K., Kirschner J., Venhoff A.C., Walker U.A. (2010). Oral uridine supplementation antagonizes the peripheral neuropathy and encephalopathy induced by antiretroviral nucleoside analogues. AIDS.

[143] Ewings E.L., Gerschenson M., St Claire M.C., Nagashima K., Skopets B., Harbaugh S.W., Harbaugh J.W., Poirier M.C. (2000). Genotoxic and functional consequences of transplacental zidovudine exposure in fetal monkey brain mitochondria. J. Acquir. Immune Defic. Syndr..

[144] Haik S., Gauthier L.R., Granotier C., Peyrin J.M., Lages C.S., Dormont D., Boussin F.D. (2000). Fibroblast growth factor 2 up regulates telomerase activity in neural precursor cells. Oncogene.

[145] Sanchez A.B., Varano G.P., de Rozieres C.M., Maung R., Catalan I.C., Dowling C.C., Sejbuk N.E., Hoefer M.M., Kaul M. (2016). Antiretrovirals, methamphetamine, and HIV-1 envelope protein gp120 compromise neuronal energy homeostasis in association with various degrees of synaptic and neuritic damage. Antimicrob. Agents Chemother..

[146] Floris-Moore M.A., Mollan K., Wilkin A.M., Johnson M.A., Kashuba A.D., Wohl D.A., Patterson K.B., Francis O., Kronk C., Eron J.J. (2016). Antiretroviral activity and safety of once-daily etravirine in treatment-naive HIV-infected adults: 48-week results. Antivir. Ther..

[147] Brown L.A., Jin J., Ferrell D., Sadic E., Obregon D., Smith A.J., Tan J., Giunta B. (2014). Efavirenz promotes beta-secretase expression and increased abeta1–40,42 via oxidative stress and reduced microglial phagocytosis: Implications for hiv associated neurocognitive disorders (hand). PLoS ONE.

[148] Decloedt E.H., Maartens G. (2013). Neuronal toxicity of efavirenz: A systematic review. Expert Opin. Drug Saf..

[149] Purnell P.R., Fox H.S. (2014). Efavirenz induces neuronal autophagy and mitochondrial alterations. J. Pharmacol. Exp. Ther..

[150] Blas-Garcia A., Polo M., Alegre F., Funes H.A., Martinez E., Apostolova N., Esplugues J.V. (2014). Lack of mitochondrial toxicity of darunavir, raltegravir and rilpivirine in neurons and hepatocytes: A comparison with efavirenz. J. Antimicrob. Chemother..

[151] Bertrand L., Toborek M. (2015). Dysregulation of endoplasmic reticulum stress and autophagic responses by the antiretroviral drug efavirenz. Mol. Pharmacol..

[152] Gatanaga H., Hayashida T., Tsuchiya K., Yoshino M., Kuwahara T., Tsukada H., Fujimoto K., Sato I., Ueda M., Horiba M. (2007). Successful efavirenz dose reduction in HIV type 1-infected individuals with cytochrome p450 2b6 *6 and *26. Clin. Infect. Dis..

[153] Guidelines for the Use of Antiretroviral Agents in HIV-1-Infected Adults and Adolescents. http://aidsinfo.nih.gov/contentfiles/lvguidelines/AdultandAdolescentGL.pdf.

[154] Bonfanti P., Valsecchi L., Parazzini F., Carradori S., Pusterla L., Fortuna P., Timillero L., Alessi F., Ghiselli G., Gabbuti A. (2000). Incidence of adverse reactions in HIV patients treated with protease inhibitors: A cohort study. Coordinamento italiano studio allergia e infezione da HIV (CISAI) group. J. Acquir. Immune Defic. Syndr..

[155] Ingelman-Sundberg M. (2004). Pharmacogenetics of cytochrome p450 and its applications in drug therapy: The past, present and future. Trends Pharmacol. Sci..

[156] Gonzalez-Baeza A., Carvajal F., Bayon C., Perez-Valero I., Estebanez M., Bernardino J.I., Monge S., Lagarde M., Hernando A., Arnalich F. (2014). Pattern of neurocognitive function in patients receiving boosted protease inhibitor monotherapy: A detailed neuropsychological study. J. Neurovirol..

[157] James C.W., McNelis K.C., Matalia M.D., Cohen D.M., Szabo S. (2002). Central nervous system toxicity and amprenavir oral solution. Ann. Pharmacother..

[158] Pettersen J.A., Jones G., Worthington C., Krentz H.B., Keppler O.T., Hoke A., Gill M.J., Power C. (2006). Sensory neuropathy in human immunodeficiency virus/acquired immunodeficiency syndrome patients: Protease inhibitor-mediated neurotoxicity. Ann. Neurol..

[159] Vivithanaporn P., Asahchop E.L., Acharjee S., Baker G.B., Power C. (2016). HIV protease inhibitors disrupt astrocytic glutamate transporter function and neurobehavioral performance. AIDS.

[160] Gannon P.J., Akay-Espinoza C., Yee A.C., Briand L.A., Erickson M.A., Gelman B.B., Gao Y., Haughey N.J., Zink M.C., Clements J.E. (2017). HIV protease inhibitors alter amyloid precursor protein processing via beta-site amyloid precursor protein cleaving enzyme-1 translational up-regulation. Am. J. Pathol..

[161] Cohen C., Elion R., Ruane P., Shamblaw D., DeJesus E., Rashbaum B., Chuck S.L., Yale K., Liu H.C., Warren D.R. (2011). Randomized, phase 2 evaluation of two single-tablet regimens elvitegravir/cobicistat/ emtricitabine/tenofovir disoproxil fumarate versus efavirenz/emtricitabine/tenofovir disoproxil fumarate for the initial treatment of HIV infection. AIDS.

[162] Harris M., Larsen G., Montaner J.S. (2008). Exacerbation of depression associated with starting raltegravir: A report of four cases. AIDS.

[163] Teppler H., Brown D.D., Leavitt R.Y., Sklar P., Wan H., Xu X., Lievano F., Lehman H.P., Mast T.C., Nguyen B.Y. (2011). Long-term safety from the raltegravir clinical development program. Curr. HIV Res..

[164] Fung H.B., Guo Y. (2004). Enfuvirtide: A fusion inhibitor for the treatment of hiv infection. Clin. Ther..

[165] Lalezari J.P., Henry K., O’Hearn M., Montaner J.S., Piliero P.J., Trottier B., Walmsley S., Cohen C., Kuritzkes D.R., Eron J.J. (2003). Enfuvirtide, an HIV-1 fusion inhibitor, for drug-resistant HIV infection in north and South America. N. Engl. J. Med..

[166] Cherry C.L., Duncan A.J., Mackie K.F., Wesselingh S.L., Brew B.J. (2008). A report on the effect of commencing enfuvirtide on peripheral neuropathy. AIDS Res. Hum. Retrovir..

[167] Lazzarin A., Clotet B., Cooper D., Reynes J., Arasteh K., Nelson M., Katlama C., Stellbrink H.J., Delfraissy J.F., Lange J. (2003). Efficacy of enfuvirtide in patients infected with drug-resistant HIV-1 in europe and australia. N. Engl. J. Med..

[168] Garvey L., Nelson M., Latch N., Erlwein O.W., Allsop J.M., Mitchell A., Kaye S., Watson V., Back D., Taylor-Robinson S.D. (2012). CNS effects of a CCR5 inhibitor in HIV-infected subjects: A pharmacokinetic and cerebral metabolite study. J. Antimicrob. Chemother..

[169] Boesecke C., Pett S.L. (2012). Clinical studies with chemokine receptor-5 (CCR5)-inhibitors. Curr. Opin. HIV AIDS.

[170] Kelly K.M., Beck S.E., Pate K.A., Queen S.E., Dorsey J.L., Adams R.J., Avery L.B., Hubbard W., Tarwater P.M., Mankowski J.L. (2013). Neuroprotective maraviroc monotherapy in simian immunodeficiency virus-infected macaques: Reduced replicating and latent SIV in the brain. AIDS.

[171] Murray K.J., Grom A.A., Thompson S.D., Lieuwen D., Passo M.H., Glass D.N. (1998). Contrasting cytokine profiles in the synovium of different forms of juvenile rheumatoid arthritis and juvenile spondyloarthropathy: Prominence of interleukin 4 in restricted disease. J. Rheumatol..

[172] Barr A.M., Panenka W.J., MacEwan G.W., Thornton A.E., Lang D.J., Honer W.G., Lecomte T. (2006). The need for speed: An update on methamphetamine addiction. J. Psychiatry Neurosci..

[173] Kaye S., McKetin R., Duflou J., Darke S. (2007). Methamphetamine and cardiovascular pathology: A review of the evidence. Addiction.

[174] Sekine Y., Ouchi Y., Sugihara G., Takei N., Yoshikawa E., Nakamura K., Iwata Y., Tsuchiya K.J., Suda S., Suzuki K. (2008). Methamphetamine causes microglial activation in the brains of human abusers. J. Neurosci..

[175] Jaehne E.J., Salem A., Irvine R.J. (2007). Pharmacological and behavioral determinants of cocaine, methamphetamine, 3,4-methylenedioxymethamphetamine, and para-methoxyamphetamine-induced hyperthermia. Psychopharmacology.

[176] Albertson T.E., Derlet R.W., Van Hoozen B.E. (1999). Methamphetamine and the expanding complications of amphetamines. West. J. Med..

[177] Murray J.B. (1998). Psychophysiological aspects of amphetamine-methamphetamine abuse. J. Psychol..

[178] Scott J.C., Woods S.P., Matt G.E., Meyer R.A., Heaton R.K., Atkinson J.H., Grant I. (2007). Neurocognitive effects of methamphetamine: A critical review and meta-analysis. Neuropsychol. Rev..

[179] Theodore S., Cass W.A., Nath A., Maragos W.F. (2007). Progress in understanding basal ganglia dysfunction as a common target for methamphetamine abuse and HIV-1 neurodegeneration. Curr. HIV Res..

[180] Lynch J., House M.A. (1992). Cardiovascular effects of methamphetamine. J. Cardiovasc. Nurs..

[181] Perez J.A., Arsura E.L., Strategos S. (1999). Methamphetamine-related stroke: Four cases. J. Emerg. Med..

[182] McCann U.D., Wong D.F., Yokoi F., Villemagne V., Dannals R.F., Ricaurte G.A. (1998). Reduced striatal dopamine transporter density in abstinent methamphetamine and methcathinone users: Evidence from positron emission tomography studies with [11c]win-35,428. J. Neurosci..

[183] Wilson J.M., Kalasinsky K.S., Levey A.I., Bergeron C., Reiber G., Anthony R.M., Schmunk G.A., Shannak K., Haycock J.W., Kish S.J. (1996). Striatal dopamine nerve terminal markers in human, chronic methamphetamine users. Nat. Med..

[184] Volkow N.D., Chang L., Wang G.J., Fowler J.S., Ding Y.S., Sedler M., Logan J., Franceschi D., Gatley J., Hitzemann R. (2001). Low level of brain dopamine d2 receptors in methamphetamine abusers: Association with metabolism in the orbitofrontal cortex. Am. J. Psychiatry.

[185] Sekine Y., Ouchi Y., Takei N., Yoshikawa E., Nakamura K., Futatsubashi M., Okada H., Minabe Y., Suzuki K., Iwata Y. (2006). Brain serotonin transporter density and aggression in abstinent methamphetamine abusers. Arch. Gen. Psychiatry.

[186] Volkow N.D., Chang L., Wang G.J., Fowler J.S., Franceschi D., Sedler M.J., Gatley S.J., Hitzemann R., Ding Y.S., Wong C. (2001). Higher cortical and lower subcortical metabolism in detoxified methamphetamine abusers. Am. J. Psychiatry.

[187] Cass W.A. (1997). Decreases in evoked overflow of dopamine in rat striatum after neurotoxic doses of methamphetamine. J. Pharmacol. Exp. Ther..

[188] Thompson P.M., Hayashi K.M., Simon S.L., Geaga J.A., Hong M.S., Sui Y., Lee J.Y., Toga A.W., Ling W., London E.D. (2004). Structural abnormalities in the brains of human subjects who use methamphetamine. J. Neurosci..

[189] Thomas D.M., Kuhn D.M. (2005). Attenuated microglial activation mediates tolerance to the neurotoxic effects of methamphetamine. J. Neurochem..

[190] Ricaurte G.A., Guillery R.W., Seiden L.S., Schuster C.R., Moore R.Y. (1982). Dopamine nerve terminal degeneration produced by high doses of methylamphetamine in the rat brain. Brain Res..

[191] Fukui K., Nakajima T., Kariyama H., Kashiba A., Kato N., Tohyama I., Kimura H. (1989). Selective reduction of serotonin immunoreactivity in some forebrain regions of rats induced by acute methamphetamine treatment; quantitative morphometric analysis by serotonin immunocytochemistry. Brain Res..

[192] Hotchkiss A.J., Gibb J.W. (1980). Long-term effects of multiple doses of methamphetamine on tryptophan hydroxylase and tyrosine hydroxylase activity in rat brain. J. Pharmacol. Exp. Ther..

[193] Wagner G.C., Ricaurte G.A., Seiden L.S., Schuster C.R., Miller R.J., Westley J. (1980). Long-lasting depletions of striatal dopamine and loss of dopamine uptake sites following repeated administration of methamphetamine. Brain Res..

[194] Nakayama M., Koyama T., Yamashita I. (1993). Long-lasting decrease in dopamine uptake sites following repeated administration of methamphetamine in the rat striatum. Brain Res..

[195] Hirata H., Ladenheim B., Rothman R.B., Epstein C., Cadet J.L. (1995). Methamphetamine-induced serotonin neurotoxicity is mediated by superoxide radicals. Brain Res..

[196] Deng X., Cadet J.L. (1999). Methamphetamine administration causes overexpression of nnos in the mouse striatum. Brain Res..

[197] Frey K., Kilbourn M., Robinson T. (1997). Reduced striatal vesicular monoamine transporters after neurotoxic but not after behaviorally-sensitizing doses of methamphetamine. Eur. J. Pharmacol..

[198] Eisch A.J., Marshall J.F. (1998). Methamphetamine neurotoxicity: Dissociation of striatal dopamine terminal damage from parietal cortical cell body injury. Synapse.

[199] O’Dell S.J., Marshall J.F. (2000). Repeated administration of methamphetamine damages cells in the somatosensory cortex: Overlap with cytochrome oxidase-rich barrels. Synapse.

[200] Schmued L.C., Bowyer J.F. (1997). Methamphetamine exposure can produce neuronal degeneration in mouse hippocampal remnants. Brain Res..

[201] Deng X., Ladenheim B., Jayanthi S., Cadet J.L. (2007). Methamphetamine administration causes death of dopaminergic neurons in the mouse olfactory bulb. Biol. Psychiatry.

[202] Jayanthi S., Deng X., Ladenheim B., McCoy M.T., Cluster A., Cai N.S., Cadet J.L. (2005). Calcineurin/NFAT-induced up-regulation of the Fas ligand/Fas death pathway is involved in methamphetamine-induced neuronal apoptosis. Proc. Natl. Acad. Sci. USA.

[203] Thiriet N., Deng X., Solinas M., Ladenheim B., Curtis W., Goldberg S.R., Palmiter R.D., Cadet J.L. (2005). Neuropeptide y protects against methamphetamine-induced neuronal apoptosis in the mouse striatum. J. Neurosci..

[204] Seiden L.S., Sabol K.E., Ricaurte G.A. (1993). Amphetamine: Effects on catecholamine systems and behavior. Annu. Rev. Pharmacol. Toxicol..

[205] Erickson J.D., Eiden L.E. (1993). Functional identification and molecular cloning of a human brain vesicle monoamine transporter. J. Neurochem..

[206] Sulzer D., Sonders M.S., Poulsen N.W., Galli A. (2005). Mechanisms of neurotransmitter release by amphetamines: A review. Prog. Neurobiol..

[207] Nash J.F., Yamamoto B.K. (1992). Methamphetamine neurotoxicity and striatal glutamate release: Comparison to 3,4-methylenedioxymethamphetamine. Brain Res..

[208] Yu Q., Zhang D., Walston M., Zhang J., Liu Y., Watson R.R. (2002). Chronic methamphetamine exposure alters immune function in normal and retrovirus-infected mice. Int. Immunopharmacol..

[209] In S.W., Son E.W., Rhee D.K., Pyo S. (2005). Methamphetamine administration produces immunomodulation in mice. J. Toxicol. Environ. Health A.

[210] Talloczy Z., Martinez J., Joset D., Ray Y., Gacser A., Toussi S., Mizushima N., Nosanchuk J., Goldstein H., Loike J. (2008). Methamphetamine inhibits antigen processing, presentation, and phagocytosis. PLoS Pathog..

[211] Krasnova I.N., Cadet J.L. (2009). Methamphetamine toxicity and messengers of death. Brain Res. Rev..

[212] Shah A., Kumar S., Simon S.D., Singh D.P., Kumar A. (2013). HIV gp120- and methamphetamine-mediated oxidative stress induces astrocyte apoptosis via cytochrome p450 2e1. Cell Death Dis..

[213] Carvalho B.M., Guadagnini D., Tsukumo D.M., Schenka A.A., Latuf-Filho P., Vassallo J., Dias J.C., Kubota L.T., Carvalheira J.B., Saad M.J. (2012). Modulation of gut microbiota by antibiotics improves insulin signalling in high-Fat fed mice. Diabetologia.

[214] Vearrier D., Greenberg M.I., Miller S.N., Okaneku J.T., Haggerty D.A. (2012). Methamphetamine: History, pathophysiology, adverse health effects, current trends, and hazards associated with the clandestine manufacture of methamphetamine. Dis. Mon..

[215] Kanthasamy A., Anantharam V., Ali S.F., Kanthasamy A.G. (2006). Methamphetamine induces autophagy and apoptosis in a mesencephalic dopaminergic neuronal culture model: Role of cathepsin-D in methamphetamine-induced apoptotic cell death. Ann. N. Y. Acad. Sci..

[216] Larsen K.E., Fon E.A., Hastings T.G., Edwards R.H., Sulzer D. (2002). Methamphetamine-induced degeneration of dopaminergic neurons involves autophagy and upregulation of dopamine synthesis. J. Neurosci..

[217] Pasquali L., Lazzeri G., Isidoro C., Ruggieri S., Paparelli A., Fornai F. (2008). Role of autophagy during methamphetamine neurotoxicity. Ann. N. Y. Acad. Sci..

[218] Yu S., Zhu L., Shen Q., Bai X., Di X. (2015). Recent advances in methamphetamine neurotoxicity mechanisms and its molecular pathophysiology. Behav. Neurol..

[219] Soontornniyomkij V., Kesby J.P., Morgan E.E., Bischoff-Grethe A., Minassian A., Brown G.G., Grant I., Translational Methamphetamine AIDS Research Center (TMARC) Group (2016). Effects of hiv and methamphetamine on brain and behavior: Evidence from human studies and animal models. J. Neuroimmune Pharmacol..

[220] Marquez C., Mitchell S.J., Hare C.B., John M., Klausner J.D. (2009). Methamphetamine use, sexual activity, patient-provider communication, and medication adherence among HIV-infected patients in care, San Francisco 2004–2006. AIDS Care.

[221] Langford D., Adame A., Grigorian A., Grant I., McCutchan J.A., Ellis R.J., Marcotte T.D., Masliah E. (2003). Patterns of selective neuronal damage in methamphetamine-user AIDS patients. J. Acquir. Immune Defic. Syndr..

[222] Roberts A.J., Maung R., Sejbuk N.E., Ake C., Kaul M. (2010). Alteration of methamphetamine-induced stereotypic behaviour in transgenic mice expressing HIV-1 envelope protein gp120. J. Neurosci. Methods.

[223] Pendyala G., Buescher J.L., Fox H.S. (2012). Methamphetamine and inflammatory cytokines increase neuronal na+/k+-atpase isoform 3: Relevance for HIV associated neurocognitive disorders. PLoS ONE.

[224] Bortell N., Morsey B., Basova L., Fox H.S., Marcondes M.C. (2015). Phenotypic changes in the brain of SIV-infected macaques exposed to methamphetamine parallel macrophage activation patterns induced by the common gamma-chain cytokine system. Front. Microbiol..

[225] Hoefer M.M., Sanchez A.B., Maung R., de Rozieres C.M., Catalan I.C., Dowling C.C., Thaney V.E., Pina-Crespo J., Zhang D., Roberts A.J. (2015). Combination of methamphetamine and HIV-1 gp120 causes distinct long-term alterations of behavior, gene expression, and injury in the central nervous system. Exp. Neurol..

[226] Almeida A., Almeida J., Bolanos J.P., Moncada S. (2001). Different responses of astrocytes and neurons to nitric oxide: The role of glycolytically generated atp in astrocyte protection. Proc. Natl. Acad. Sci. USA.

[227] Almeida A., Moncada S., Bolanos J.P. (2004). Nitric oxide switches on glycolysis through the AMP protein kinase and 6-phosphofructo-2-kinase pathway. Nat. Cell Biol..

[228] Hung K.M., Chen P.C., Hsieh H.C., Calkins M.J. (2017). Mitochondrial defects arise from nucleoside/nucleotide reverse transcriptase inhibitors in neurons: Potential contribution to HIV-associated neurocognitive disorders. Biochim. Biophys. Acta.

[229] Wong-Riley M.T. (1989). Cytochrome oxidase: An endogenous metabolic marker for neuronal activity. Trends Neurosci..

[230] Maday S., Twelvetrees A.E., Moughamian A.J., Holzbaur E.L. (2014). Axonal transport: Cargo-specific mechanisms of motility and regulation. Neuron.

[231] Cai Q., Zakaria H.M., Simone A., Sheng Z.H. (2012). Spatial parkin translocation and degradation of damaged mitochondria via mitophagy in live cortical neurons. Curr. Biol..

[232] Palikaras K., Tavernarakis N. (2012). Mitophagy in neurodegeneration and aging. Front. Genet..

[233] Pickrell A.M., Youle R.J. (2015). The roles of PINK1, parkin, and mitochondrial fidelity in parkinson’s disease. Neuron.

[234] Hardie D.G. (2011). Amp-activated protein kinase: An energy sensor that regulates all aspects of cell function. Genes Dev..

[235] Oakhill J.S., Steel R., Chen Z.P., Scott J.W., Ling N., Tam S., Kemp B.E. (2011). Ampk is a direct adenylate charge-regulated protein kinase. Science.

[236] Laplante M., Sabatini D.M. (2012). Mtor signaling in growth control and disease. Cell.

[237] Mizushima N., Levine B., Cuervo A.M., Klionsky D.J. (2008). Autophagy fights disease through cellular self-digestion. Nature.

[238] Fields J., Dumaop W., Rockenstein E., Mante M., Spencer B., Grant I., Ellis R., Letendre S., Patrick C., Adame A. (2013). Age-dependent molecular alterations in the autophagy pathway in hive patients and in a gp120 tg mouse model: Reversal with beclin-1 gene transfer. J. Neurovirol..

[239] Balgi A.D., Fonseca B.D., Donohue E., Tsang T.C., Lajoie P., Proud C.G., Nabi I.R., Roberge M. (2009). Screen for chemical modulators of autophagy reveals novel therapeutic inhibitors of mtorc1 signaling. PLoS ONE.

[240] Liu B., Liu X., Tang S.J. (2016). Interactions of opioids and hiv infection in the pathogenesis of chronic pain. Front. Microbiol..

[241] Dhillon N.K., Williams R., Peng F., Tsai Y.J., Dhillon S., Nicolay B., Gadgil M., Kumar A., Buch S.J. (2007). Cocaine-mediated enhancement of virus replication in macrophages: Implications for human immunodeficiency virus-associated dementia. J. Neurovirol..

[242] Liang H., Wang X., Chen H., Song L., Ye L., Wang S.H., Wang Y.J., Zhou L., Ho W.Z. (2008). Methamphetamine enhances hiv infection of macrophages. Am. J. Pathol..

[243] Wang Y., Wang X., Ye L., Li J., Song L., Fulambarkar N., Ho W. (2012). Morphine suppresses IFN signaling pathway and enhances AIDS virus infection. PLoS ONE.

[244] Rivera-Rivera L., Perez-Laspiur J., Colon K., Melendez L.M. (2011). Inhibition of interferon response by cystatin b: Implication in hiv replication of macrophage reservoirs. J. Neurovirol..

[245] Purohit V., Rapaka R.S., Rutter J., Shurtleff D. (2012). Do opioids activate latent HIV-1 by down-regulating anti-HIV micrornas?. J. Neuroimmune Pharmacol..

[246] Sampey G.C., Meyering S.S., Asad Z.M., Saifuddin M., Hakami R.M., Kashanchi F. (2014). Exosomes and their role in CNS viral infections. J. Neurovirol..

[247] Hu G., Yao H., Chaudhuri A.D., Duan M., Yelamanchili S.V., Wen H., Cheney P.D., Fox H.S., Buch S. (2012). Exosome-mediated shuttling of microrna-29 regulates HIV tat and morphine-mediated neuronal dysfunction. Cell Death Dis..

[248] Rahimian P., He J.J. (2016). Exosome-associated release, uptake, and neurotoxicity of HIV-1 tat protein. J. Neurovirol..

[249] Raymond A.D., Diaz P., Chevelon S., Agudelo M., Yndart-Arias A., Ding H., Kaushik A., Jayant R.D., Nikkhah-Moshaie R., Roy U. (2016). Microglia-derived hiv NEF+ exosome impairment of the blood-brain barrier is treatable by nanomedicine-based delivery of nef peptides. J. Neurovirol..

